# Brief Monocular Deprivation as an Assay of Short-Term Visual Sensory Plasticity in Schizophrenia – “The Binocular Effect”

**DOI:** 10.3389/fpsyt.2013.00164

**Published:** 2013-12-17

**Authors:** John J. Foxe, Sherlyn Yeap, Victoria M. Leavitt

**Affiliations:** ^1^The Cognitive Neurophysiology Laboratory, Nathan S. Kline Institute for Psychiatric Research, Orangeburg, NY, USA; ^2^The Cognitive Neurophysiology Laboratory, St. Vincent’s Hospital, Dublin, Ireland; ^3^Program in Neuropsychology, Department of Psychology, Queens College, The City University of New York, Flushing, NY, USA; ^4^The Sheryl and Daniel R. Tishman Cognitive Neurophysiology Laboratory, Departments of Pediatrics and Neuroscience, Children’s Evaluation and Rehabilitation Center (CERC), Albert Einstein College of Medicine, Bronx, NY, USA

**Keywords:** EEG, psychosis, visual evoked potential, event-related potential, endophenotype, genetic liability, biomarker, vision

## Abstract

**Background**: Visual sensory processing deficits are consistently observed in schizophrenia, with clear amplitude reduction of the visual evoked potential (VEP) during the initial 50–150 ms of processing. Similar deficits are seen in unaffected first-degree relatives and drug-naïve first-episode patients, pointing to these deficits as potential endophenotypic markers. Schizophrenia is also associated with deficits in neural plasticity, implicating dysfunction of both glutamatergic and GABAergic systems. Here, we sought to understand the intersection of these two domains, asking whether short-term plasticity during early visual processing is specifically affected in schizophrenia.

**Methods**: Brief periods of monocular deprivation (MD) induce relatively rapid changes in the amplitude of the early VEP – i.e., short-term plasticity. Twenty patients and 20 non-psychiatric controls participated. VEPs were recorded during binocular viewing, and were compared to the sum of VEP responses during brief monocular viewing periods (i.e., Left-eye + Right-eye viewing).

**Results**: Under monocular conditions, neurotypical controls exhibited an effect that patients failed to demonstrate. That is, the amplitude of the summed monocular VEPs was robustly greater than the amplitude elicited binocularly during the initial sensory processing period. In patients, this “binocular effect” was absent.

**Limitations**: Patients were all medicated. Ideally, this study would also include first-episode unmedicated patients.

**Conclusion**: These results suggest that short-term compensatory mechanisms that allow healthy individuals to generate robust VEPs in the context of MD are not effectively activated in patients with schizophrenia. This simple assay may provide a useful biomarker of short-term plasticity in the psychotic disorders and a target endophenotype for therapeutic interventions.

## Introduction

Visual sensory processing deficits have been consistently documented in schizophrenia using the visual evoked potential (VEP) method (1–7). These deficits, which manifest as substantial reduction in amplitude of the early P1 component of the VEP, represent a potentially promising endophenotypic marker insofar as healthy first-degree biological relatives of schizophrenia patients show entirely similar attenuation of the P1 (8). Likewise, robust deficits are seen in first-episode patients and in young adults with high schizotypy, supporting the notion that visual sensory processing deficits may be related to predisposing genetic risk for the disorder rather than progression of the disease state itself (9–12). The relationship of visual sensory deficits to genetic liability was further emphasized in a pair of studies linking them to specific risk haplotypes for schizophrenia on the dysbindin gene (DTNBP1) (13) and the nitric oxide synthasase-1 gene (NOS1) (14). Taken together, these studies point to early visual sensory processing deficits as a potentially valuable tool in early detection efforts and to the possibility that this biomarker might expand our abilities to identify children at risk during the prodromal stages of the disorder (15, 16).

Dysfunctional neural plasticity is another key aspect of cortical processing implicated in the pathophysiology of schizophrenia (17, 18). Recently, Cavus and colleagues assessed visual cortical plasticity in schizophrenia using an experimental design believed to be a non-invasive analog of long-term potentiation (LTP) studies conducted in animal models (18). They used high-frequency visual stimulation (∼9 Hz) as a form of photic tetanus, as a proxy for the tetanic electrical inputs used to induce LTP in animals, since prior work had shown that the amplitude of early VEP components was enhanced following high-frequency photic inputs (19). Cavus showed that the initial C1 component of the VEP was indeed enhanced in neurotypical adults following photic tetanus, but no such enhancement was seen in a group of patients with schizophrenia. In line with the well-established role of the glutamatergic system in LTP induction in animals, they interpreted their results as evidence for dysfunctional *N*-methyl-d-aspartate (NMDA) receptor-mediated plasticity in schizophrenia.

Given the existing evidence for visual sensory processing deficits and emerging evidence for deficits in visual sensory plasticity, we set out here to more fully explore the intersection of these two candidate endophenotypes. We asked whether short-term visual plasticity is compromised in schizophrenia and whether deficits in plasticity would be related to more fundamental deficits of sensory transmission. Our measure of plasticity was based on early work by Tyler and Kaitz (20), who used a monocular deprivation (MD) paradigm to induce short-term changes in VEP amplitude. These authors showed that by covering one eye for several hours, an increase in VEP amplitude was observed across time for stimulation of the non-occluded eye. That is, immediately following eye-occlusion, the monocularly derived VEP amplitude attenuates relative to binocular viewing. This is unsurprising, of course, since half of the afferent input to the system has been removed. However, over the following 2–6 h, the monocularly derived VEP gained significantly in amplitude, suggesting relatively short-term reorganization of early visual cortical representations to compensate for the missing input. There is also considerable evidence in the literature for the non-linear nature of binocular interactions in humans (21–23). That is, when VEPs elicited from monocular viewing are summed together, the resultant “synthetic” response is found to be of greater amplitude than the VEP evoked during binocular viewing (i.e., Left-eye alone + Right-eye alone > Both eyes together). The implication is that during binocular viewing, there is substantial interaction between monocular dominance columns, likely due to cross-inhibitory mechanisms (24). Thus, this paradigm likely assays short-term plasticity that is mainly driven by changes in GABAergic mechanisms, unlike the study of Cavus and colleagues where NMDA functioning was the hypothesized synaptic substrate (18).

There is also good reason to expect that there might be specific impairment of such binocular interactions in schizophrenia (25). Ocular dominance columns in visual cortex segregate cells that are selective for left-eye, right-eye, or binocular stimuli (26). While the exact functional purpose of this columnar organization is not fully known, it is highly likely that one of its functions is in the computation of stereopsis, or depth perception, and patients with schizophrenia show a marked deficit in binocular depth perception (25), suggesting that interaction of ocular dominance columns may indeed be compromised.

Here, we utilized an MD paradigm similar to that of Tyler and Kaitz (20), though a substantially abbreviated version that allowed us to capture dynamic effects of MD in just 25 min. Developing tests that are easily administered over a relatively short period will be essential if these tests are to have genuine clinical utility. VEPs were recorded from participants while they viewed simple isolated-check stimuli both binocularly and monocularly. The predictions were straightforward. Once input to one or the other eye is occluded (using a simple eye patch), the expectation is that early VEP amplitude elicited from single-eye viewing will diminish immediately thereafter, but that a relatively rapid recovery of amplitude will be evident in the following minutes as the visual system compensates for missing input. Our proposition is that this recovery of amplitude results from a basic release from the inter-ocular inhibition inherent in binocular interactions, and that dysregulation of this short-term plasticity in schizophrenia will result in an attenuated VEP. As such, our main hypothesis is that the rapid recovery of VEPs exhibited by healthy controls under conditions of MD will be substantially attenuated in patients with schizophrenia.

## Materials and Methods

The procedures of this study were approved by the Ethics Committee of St. Vincent’s Hospital in Fairview, Dublin, and the Institutional Review Board of the City University of New York. All procedures were consistent with the ethical standards laid out in the Declaration of Helsinki. Participants received a modest fee for taking part in the study.

### Participants

Informed consent was obtained from 20 (4 female) patients with schizophrenia, aged 28–63 (mean = 41.6 ± 12.2 years), from the St. Vincent’s Hospital Catchment Area in Fairview, Dublin. The mean score on the Brief Psychiatric Rating Scale (BPRS) for the patients was 39.5 ± 8.6 and for the Scale for the Assessment of Negative Symptoms (SANS) was 33.1 ± 16.8. Control participants comprised 20 (4 female) volunteers aged 19–61 (mean = 39.0 ± 15.2 years). The mean age of patients and controls did not differ significantly (*p* = 0.48). Seventeen of the 20 patients and 18 of the 20 controls were right-handed as assessed by the Edinburgh Handedness Inventory (27). All subjects reported normal or corrected-to-normal vision; where possible, this was corroborated by medical records. Controls were free of any psychiatric illness or symptoms by self-report using criteria from the SCID-NP (28).

Patients met the following inclusion/exclusion criteria: inclusion: (1) current DSM-IV-defined diagnosis of schizophrenia. A best estimate diagnostic approach was utilized in which information from the Structured Clinical Interview for DSM-IV Axis I Disorders (SCID-I/P) (28) was supplemented by information from family members, psychiatrists, and medical records to generate a diagnosis, (2) aged between 18 and 65, (3) any race, (4) male or female, (5) competent and willing to sign informed consent. Exclusion: (1) organic brain disorder, mental retardation, or significant medical illness, (2) current substance-induced psychotic disorder or a psychotic disorder due to a general medical condition determined by DSM-IV criteria, (3) significant risk of suicidal or homicidal behavior, (4) any history of visual impairment beyond corrected-to-normal vision, (5) any additional Axis I diagnoses.

Neurotypical controls met the following inclusion/exclusion criteria: inclusion: (1) absent any current Axis I or Axis II disorder or mood or psychotic disorder for the last 5 years as assessed by the SCID-I/NP (i.e., Non-Patient version), (2) matched for age to patients with schizophrenia and between the ages of 18 and 65, (3) any race, (4) male or female; (5) competent and willing to sign informed consent, (6) no current or past history of psychotropic medication usage, (7) no family history of psychotic illness. Exclusion: (1) organic brain disorder, mental retardation, or significant medical illness, (2) significant risk of suicidal or homicidal behavior, (3) participants with prior intermittent alcohol or substance use were not excluded unless they met DSM-IV criteria for current alcohol or drug dependence in the last 6 months, (4) any history of abnormal vision beyond corrected-to-normal vision.

For all patients, demographic information was collected (see Table 1). Symptom ratings were analyzed using the BPRS and the SANS (29). Doses of anti-psychotic, anti-cholinergic, and adjuvant medications (e.g., anti-depressants, sedative/hypnotics, mood stabilizers, anti-convulsants) were recorded. Anti-psychotic doses were translated into chlorpromazine equivalents using the best available literature at the time of data analysis for conversion of dose levels of newer anti-psychotics (e.g., sertindole, seroquel). A limitation of the present study is that all patients were receiving medication at the time of testing. However, visual processing deficits have been found in both medicated and unmedicated patients [e.g., Ref. (30–33), as well as in (unmedicated) first-degree relatives of patients with schizophrenia (8)]. Medication effects, length of illness, and symptom severity should all be taken into consideration as potential confounds when conducting research with schizophrenia patients. However, evidence in the literature supports the notion that these factors do not impact VEPs generated at such early latencies as the P1 (34).

**Table 1:** **Demographic and clinical characteristics of schizophrenia patient group (*N* = 20)**.

Neuroleptics: atypical/typical/both	12/4/4
Chloropromazine (CPZ)-equivalent ± SD, daily (mg)	564 ± 423
Education ± SD (using SCID education scale 1–8)	5.1 ± 2
Illness duration ± SD	16.1 ± 9
BPRS positive symptom ± SD	39.5 ± 9
SANS total all items except global at baseline ± SD	33.1 ± 17
Visual hallucinations: past/present/none	4/1/15

### Stimuli and task

Subjects were seated in a comfortable chair in a dimly illuminated, sound-attenuated room, and asked to keep head and eye movements to a minimum. In each experimental block, subjects were presented with ∼100 isolated-check images, gray on a white background (4°× 4° visual angle) at 64% contrast and 40 line drawings of two kinds of animal (2.4° wide × 1.8° high) on a white background. A different animal pair was randomly chosen for each block, from a possible 22. This simple paradigm has been described extensively in our previous work (9) (see Figure S1 in Supplementary Material). All subjects completed nine blocks of stimulation, each lasting 3 min. These consisted of three blocks wherein the participant viewed stimuli binocularly, followed immediately by three blocks of occlusion (an eye patch) of one eye, and three blocks of occlusion of the other eye. After the first monocular series of three blocks, the patch was switched to the other eye by the experimenter and stimulus delivery begun again immediately. The order in which the first eye was occluded (i.e., right or left) was counterbalanced across participants. Stimuli were presented centrally on a computer monitor in random order, with the monitor located 160 cm directly in front of the seated subject. The timing of the presentations was such that each image appeared for 60 ms with a variable inter-stimulus interval (ISI) between 740 and 1540 ms (randomly in steps of 200 ms) during which there was a blank white screen. The target animal was displayed at the start of the task and subjects were asked to respond each time this animal was presented by pressing a button with their right thumb. They were told only to respond to target animals and to withhold responses to any other animal presented. The target and non-target animals were presented with equal probability, ensuring that a subject could not rely on the exogenous alerting nature of any non-checkerboard stimulus to respond. Furthermore, the task of discrimination was made difficult by pairing similar-looking animals (e.g., a dolphin and whale). The use of this task ensured that subjects were actively observing the stimuli. Only ERPs to the standard checkerboard stimuli were analyzed here.

### Data acquisition and statistical analysis

Continuous EEG was acquired through the ActiveTwo Biosemi™ electrode system from 72 scalp electrodes, digitized at 512 Hz with an open pass-band from DC to 150 Hz. For analysis and display purposes, data were subsequently filtered with a 0-phase-shift 40 Hz low-pass filter (24 dB/octave) and baseline corrected after acquisition. No high-pass filter was applied. With the Biosemi system, every electrode or combination of electrodes can be assigned as the “reference,” and this is done purely in software after acquisition. BioSemi replaces the “ground” electrodes used in conventional systems with two separate electrodes: common mode sense (CMS) active electrode and Driven Right Leg (DRL) passive electrode. These two electrodes form a feedback loop, which drives the average potential of the subject (the Common Mode voltage) as close as possible to the ADC reference voltage in the AD-box (the ADC reference can be considered as the amplifier “zero”). For a detailed description of the referencing and grounding conventions used by the Biosemi active electrode system, the interested reader is referred to the following website: http://www.biosemi.com/faq/cms&drl.htm. All data were re-referenced to the nasion after acquisition, for analysis. EEG was averaged off-line. Data were epoched (−100 ms pre-stimulus to 500 ms post-stimulus) and then averaged. Baseline was defined as the mean voltage over −100 to 0 ms preceding the onset of the stimulus. Trials with blinks and large eye movements were rejected off-line on the basis of horizontal and vertical electrooculogram recordings. An artifact rejection criterion of ±100 μV was used at all other electrode sites to exclude periods of high EMG and other noise-transients. From the remaining artifact-free trials, averages were computed for each participant. The average acceptance rate for the patients was 71 ± 17.6%, and for the control group 75 ± 20.0%. These averages were then visually inspected for each individual to ensure that clean recordings with sufficient numbers of trials were obtained and that no artifacts were still included. Bad channels were interpolated using BESA software. Data were ultimately averaged across all subjects (grand mean averages) within a given group (patient or control) for visual comparison at the group level and for display purposes. The reader should note that throughout this paper, we use the familiar nomenclature of the modified 10–20-electrode system to refer to the positioning of electrode sites (35).

### Analysis of the P1-deficit

We first sought to ensure that the previously established P1-deficit in schizophrenia was indeed replicated in the cohort of patients who participated here. For this analysis, only data from the binocular condition were considered. We recorded P1 peak amplitudes on an individual participant basis by surveying across six pre-determined scalp electrodes: three over the left hemiscalp (PO7/PO3/O1) and three over the right (PO8/PO4/O2). These sites were chosen for analysis based on observation of the group-averaged data (collapsed across patients and controls), since they best represented the maximal topographic distribution of the bilateral P1 component, entirely consistent with previous work (36, 37). Separate composite averages for left (PO7/PO3/O1) and right (PO8/PO4/O2) hemiscalps were computed for each subject, and peak amplitude was recorded for each hemiscalp for each subject at their individual P1 peak latency. We did not exclude any subject’s P1 on the basis of absolute amplitude; if an obvious P1-like component was discernible, its value was recorded, and included in the analysis. However, if a clearly discernible P1 was not evident over *both* hemiscalps, then data from that participant were excluded from this P1 analysis. These peak P1 amplitude data were then submitted to a mixed design ANOVA with factors of group (controls vs. patients) and scalp region (left vs. right) to compare P1 amplitudes.

### Analysis of the binocular effect

Having first established that the P1 processing deficit was evident in this cohort of schizophrenia patients, we then moved to the assessment of putative binocular interactions. Since research into binocular interaction effects using VEPs is very sparse, and the early VEP componentry has not been explicitly interrogated for such effects (to our knowledge), we had no specific way to predict precisely when during initial sensory processing we would see non-linear effects in the data as a function of binocularity. On the other hand, we reasoned that binocular interactions must surely occur during the earliest sensory processing time-frame since work from our group has shown that even trans-colossal inter-hemispheric interactions are already evident during the P1 processing time-frame (36). Thus, our approach here was to first identify the earliest binocular effect (BE) in our control cohort, thereby defining a time-window and appropriate scalp regions for subsequent between groups analyses. A clear and robust effect was indeed evident in the group-averaged data from the control group in the time-frame between 95 and 115 ms. This effect also had a bilateral occipito-parietal distribution, so average amplitude measures over the 95–115 ms time-frame were derived from the same six scalp sites (PO7/PO3/O1 over the left hemiscalp and PO8/PO4/O2 over the right) as used during the P1 analysis. Here, regardless of whether amplitude values were positive or negative during the time-frame, data from all 20 controls and all 20 patients were included, since this analysis did not depend on the identification of any particular ERP component.

The VEP BE was defined here as any deviation from the predictions of a model that assumed two independent populations of neurons whose outputs are, in the far field, simply additive. Monocular responses were added (Left-eye alone “PLUS” Right-eye alone) to yield the model’s prediction of binocularly evoked response; this trace was then subtracted from the actual binocular response to yield a difference trace which is considered to represent BE, and which we refer to as the “modulation index.” We henceforth refer to the summed waveform as SUM, and the binocularly evoked waveform as BASE.

Amplitude measures taken over the 95–115 ms time-window from the left and right hemiscalps were then submitted to a repeated measures ANOVA with between-subjects factor of group (patients vs. controls) and within-subjects factors of scalp region (left/right) and condition (SUM vs. BASE). All tests were two-tailed with a preset α-level of *p* < 0.05.

Following our primary analyses of P1 amplitude and the BE, it was of interest to further investigate spatio-temporal properties of any potential differences between groups, using the statistical cluster plot method. This procedure has been used effectively in *post hoc* analyses as a means to more fully explore complex datasets and generate pointed follow-up hypotheses (38, 39). For each group, point-wise two-tailed Student *t*-tests (between conditions) were calculated at each time-point for all electrodes, and a color map was subsequently generated marking time-points on each electrode for which the *t*-value exceeds that corresponding to a 0.05 *p*-value. Importantly, the use of this approach is limited to that of a hypothesis-generation tool. Here, it simply provided a visualization tool providing confirmatory evidence. Periods of significant difference are only plotted if an alpha criterion of <0.05 was exceeded for at least 10 consecutive data points (40).

Finally, in order to test whether a relationship between P1 amplitudes and the BE could be detected, we conducted correlation analyses. The main goal here was to assess whether those patients with the greatest P1-deficits were those who also showed the lowest binocular modulation index – that is, to assess whether the BE and P1 generation might be related. A correlation coefficient was computed for P1 amplitudes (collapsed averages for left and right hemiscalps of each subject) and modulation index values.

## Results

### Behavioral results

In the binocular, left-eye alone, and right-eye alone conditions, the mean target hit rates were all >80% and did not significantly differ between control participants and patients. These rates of performance indicate that all participants were actively observing the stimuli throughout the experimental blocks. Recall that target stimuli are not entered into the main analyses here, serving purely to maintain participants’ attention throughout the experimental blocks. No motor responses were recorded to the isolated-check stimuli that served as standards and that were explicitly analyzed in what follows.

### Replicating the visual P1-deficit

**Figure 1:**
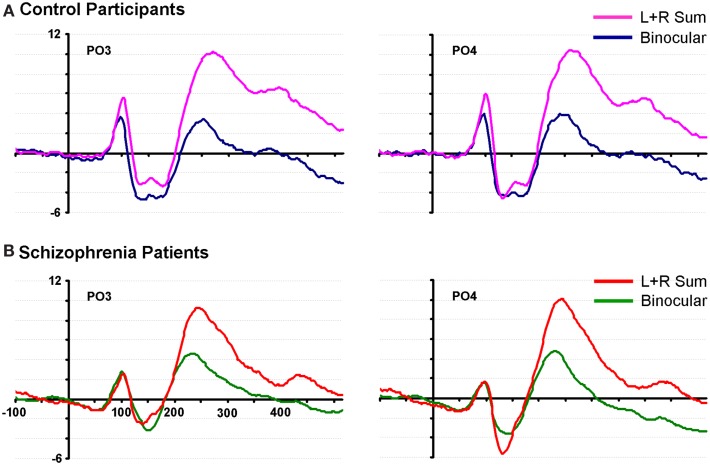
**Waveforms from two representative parieto-occipital scalp sites (PO3 and PO4) are shown for neurotypical control participants and patients with schizophrenia**. **(A)** Data from 20 control subjects are displayed in pink and blue; **(B)** data from 20 patients are displayed in red and green.

We begin with an analysis of the P1 component. The P1 peak for the control group in the binocular condition was found at ∼93 ms, and was maximal over bilateral parieto-occipital and occipital regions with entirely typical topography (37). In patients, the P1 peak was found at 97 ms over the same regions (see Figure 1).

A mixed 2 × 2 ANOVA was conducted with a between-subjects factor of group (patients vs. controls) and a within-subjects factor of hemiscalp (left vs. right). The P1 peak was identified at each site for each participant^1^, and an average of the three electrodes was calculated for each hemiscalp. A main effect of group was observed (*F*(1,31) = 7.148, *p* = 0.01), as was a significant interaction of group by hemiscalp (*F*(1,31) = 4.794, *p* = 0.036). To further examine the interaction between group and hemiscalp, protected follow-up *t*-tests were carried out for each hemiscalp separately. A significant difference was found between controls and patients over both the left (*p* = 0.01) and the right (*p* = 0.002) hemiscalps. For controls, the average P1 amplitude on the left was 5.92 ± 2.67 μV, on the right 6.72 ± 2.90. For patients, these values were 3.59 ± 2.51 (left) and 3.35 ± 2.51 (right). As such, the interaction was driven by the fact that the difference between groups was greater over the right hemiscalp than the left. Figure S2 in Supplementary Material shows a scatterplot of P1 amplitudes (collapsed across left and right hemifields) to better illustrate the group differences. A comparison of these latter values (i.e., collapsed across hemiscalps) also revealed a significant difference between groups (*t*(15) = 2.43, *p* = 0.028) with a large effect size (Cohen’s *d* = 1.05).

### The binocular effect: Characterizing inter-ocular plasticity

By visually examining the ERPs of both groups over parieto-occipital scalp, we observed a striking non-linearity in the control group for their summed VEPs (left-eye alone + right-eye alone, or SUM) compared to their binocularly evoked VEPs (BASE) (see Figure 1). We termed this non-linearity the “binocular effect.” To explore potential group differences in the BE, a 2 × 2 × 2 ANOVA was performed with factors of group (patients vs. controls), condition (BASE vs. SUM) and hemiscalp (left vs. right) for amplitude measures over the 95–115 ms time period. A significant interaction of group by condition was found (*F*(1,38) = 4.526, *p* = 0.04). Follow-up protected *t*-tests revealed a significant effect of condition over both the left and right occipital scalp in controls (*p* = 0.002 and *p* = 0.034, respectively). Patients on the other hand, failed to exhibit a significant effect of condition over either hemiscalp (*p* = 0.93 and *p* = 0.90).

**Figure 2:**
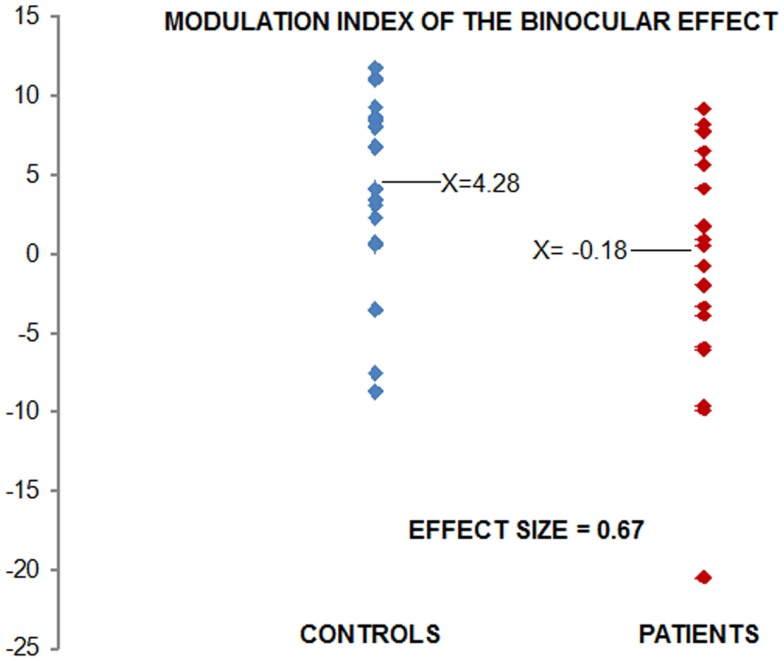
**Modulation index for the binocular effect: each point in the scatter plot represents the data for one participant**. Values were derived by subtracting Binocular (BASE) from SUM conditions. Controls are shown in blue, patients in red. Using Cohen’s *d*, the effect size of the difference is 0.67.

Figure 2 displays the modulation index. Each point on the scatterplot represents a participant’s single value for SUM minus BASE (collapsed over hemiscalps), thereby providing a snapshot of the degree to which each subject exhibited the BE. The reader will note in Figure 2 that there is one patient who could be considered an outlier, with a modulation index of approximately −20 μV. To ensure that our results were not adversely affected by inclusion of this participant’s data, we also analyzed the data excluding this participant. The main effect of group remained significant with large effect size (*d* = 0.59).

**Figure 3:**
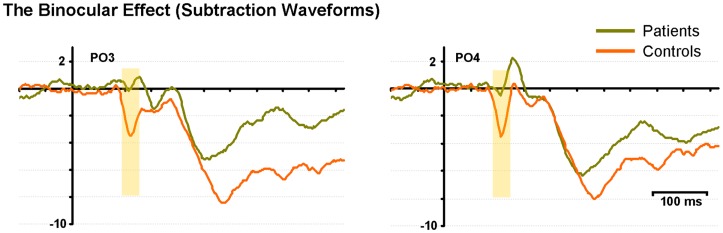
**The binocular effect is characterized with difference waveforms (SUM minus BASE) for controls (red) and patients (green) at two representative electrodes (PO3 and PO4)**. The yellow shading denotes the early “binocular effect” period.

Figure 3 displays subtraction waveforms (SUM minus BASE) to illustrate the BE in both controls and patients. These subtraction waveforms, taken from representative occipito-parietal electrode sites (PO3 and PO4) emphasize the robust BE in controls (red trace) that emerges between ∼90 and 120 ms. This difference is much less pronounced in patients (green trace). It can also be seen that later differences between SUM and BASE (from 150 ms onward), while evident in the subtraction waveforms of patients, are also of lower amplitude than those seen in controls (analyzed below).

**Figure 4:**
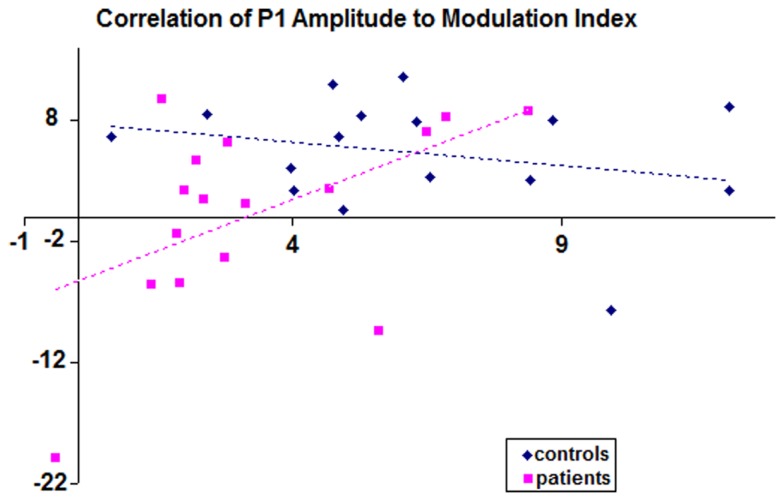
**Scatter plot is displayed showing correlations for each subject of P1 amplitude to modulation index**. The *x*-axis displays values for P1 amplitudes; *y*-axis displays binocular modulation indices. The P1 values used here are collapsed averages of left and right hemiscalp amplitudes. Controls are depicted by blue points (*n* = 16), patients by pink (*n* = 16).

Correlation analysis was conducted to assess whether a relationship between P1 amplitudes and the BE could be detected (that is, if those patients with the greatest P1-deficits were those who also showed the weakest binocular modulation index). This analysis revealed no correlation in the healthy control group (*r* = −0.26; *p* = 0.165), whereas a significant positive correlation between these two measures was found for the patients (*r* = 0.515; *p* = 0.02). Correlation scatter plots for patients and controls are overlaid in Figure 4.

Following the logic of a multivariate endophenotype as described by Price et al. (41), we combined our two metrics, P1 amplitude and the binocular modulation index, to determine whether this would result in a greater overall effect size. Our hope was that taken in concert, these measures would provide better group classification than when taken in isolation. Under this approach, the two metrics were first treated individually as independent variables, with subjects as our dependent variable. Univariate logistic regression (cut 0.5) was used to determine a regression model that maximally separated patient and control groups on the basis of each feature alone. In turn, multivariate logistic regression (cut 0.5) was then used to develop a composite regression model that was compared with the univariate models. P1 amplitude predicted group membership with 71.9% accuracy, the BE modulation index with 65.6%, and both measures taken together were 75% accurate as predictors. Full results of the analyses are displayed in Table 2.

**Table 2:** **Coefficients of univariate logistic regression models that were evaluated: univariate models for two features, then a multivariate model incorporating both features**.

Model predictor	*B*	SE	Wald statistic	df	*p*	Exp (*B*)	Overall predictor value (%)
P1 amplitude	−0.394	0.165	5.735	1	0.017	0.674	71.9
P1 constant	1.841	0.838	4.830	1	0.028	6.304	
Binoc effect	−0.139	0.073	3.585	1	0.058	0.871	65.6
Binoc effect constant	0.462	0.474	0.950	1	0.330	1.588	
Multivariate P1 amp	0.364	0.167	4.763	1	0.029	1.440	75
Multivariate binoc effect	0.127	0.081	2.460	1	0.117	1.135	

*All models were generated based on data from 16 patient and 16 control participants*.

**Figure 5:**
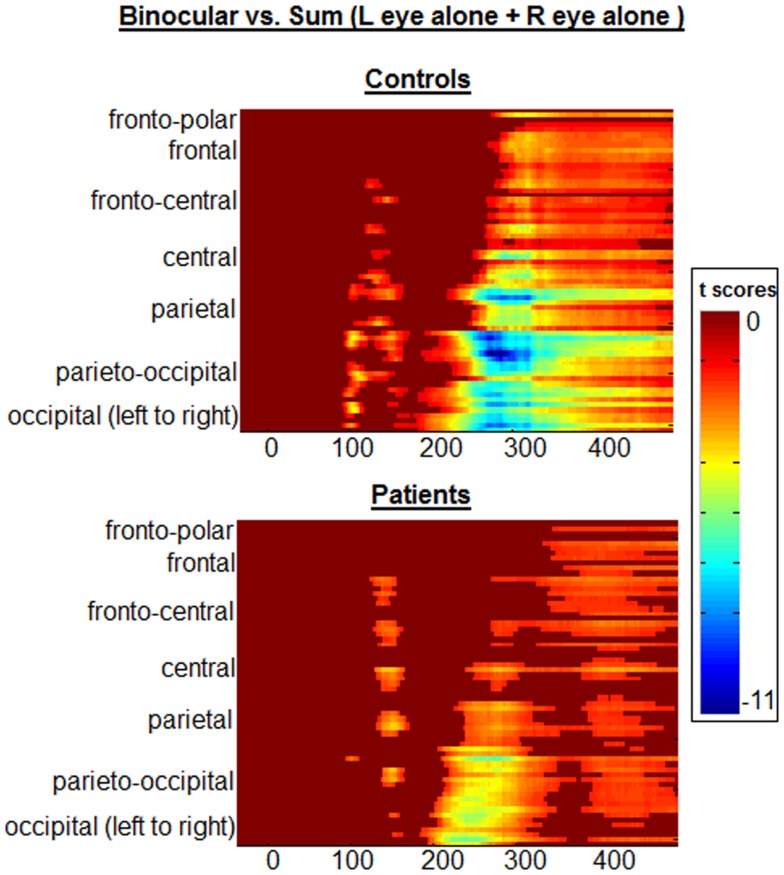
**Statistical cluster plots depict point-wise running *t*-tests comparing the amplitudes of participants’ VEPs in Binocular vs. SUM conditions**. Time with respect to stimulus onset is presented on the *x*-axis and the general topographic regions of 72 electrode positions are displayed on the *y*-axis. Color corresponds to *t* values. Periods of significant difference are only plotted if a strict alpha criterion of <0.05 was exceeded for at least 10 consecutive data points. The early “binocular effect” period has been outlined with a black box for each group.

In Figure 5, the statistical cluster plots display between-condition differences (BASE vs. SUM) for each group. Examination of these plots made clear that there were four major clusters of interest. The first of these was, of course, the one of primary interest to us, as it encapsulated the BE. This is highlighted with a black box in Figure 5. In controls, this cluster emerged at ∼90 ms, and was restricted largely to posterior scalp (i.e., parietal, parieto-occipital, and occipital regions). In patients, effects in this period were notably absent; consistent with the complete absence of any BE in patients as discussed above.

Although not directly related to our primary period of interest, we include here a full description of subsequent clusters of significance identified by this analysis. The second significance cluster emerges for both groups at ∼140–160 ms. A comparison with waveforms shows this cluster to correspond generally with the N1 component for both groups (see Figure 1), and the effect appears largely similar for both groups. A third pronounced cluster onsets at ∼200 ms; waveform comparison shows that this cluster corresponds to the first of two broad positive components elicited in both groups. Whereas this significance cluster in patients is largely confined to central, parietal, and occipital regions, in controls it may also extend more frontally. The cluster plots show that this effect is robust for both groups, although it is also clear that it is more so the case in controls. The final cluster is a much more broadly distributed effect that appears to have its onset between 200 and 300 ms and extends to the end of the sampled epoch. In controls it is less distinct as a separable cluster than in patients, but again comparing cluster plots to waveforms (Figure 1) revealed that this is largely coincident with the final positivity, onsetting between 200 and 300 ms. Again, the cluster plots show that differences between SUM and BASE waveforms are considerably more robust in controls than patients. We were interested to determine whether these later two periods would show significant group by condition interactions and therefore submitted these time-windows to a pair of *post hoc* ANOVAs. For the 200–300 ms period, a main effect was found for condition (*F*(1,38) = 78.636, *p* < 0.001), but not for group (*F*(1,38) = 0.414, *p* = 0.524). A group by condition interaction did not attain conventional levels of significance although a trend is evident (*F*(1,38) = 3.495, *p* = 0.069), and this effect may represent a good candidate for future investigation and replication. We also tested both groups for the time period 300–400 ms; here, main effects of group (*F*(1,38) = 7.774, *p* = 0.008) and condition (*F*(1,38) = 11.131, *p* = 0.002) were found, but there was no significant interaction of group by condition (*F*(1,38) = 0.883, *p* = 0.354).

## Discussion

We set out to develop a simple-to-execute test of short-term visual sensory plasticity and to assess whether this plasticity was intact in schizophrenia. A basic MD task allowed for assessment of sensory-level binocular integration. We reasoned that if patients with schizophrenia showed deficits in this plasticity, this would provide further evidence for fundamental visual sensory processing deficits in this population and might explain previous work showing deficits in depth perception (25). We first replicated the well-established P1-deficit in our patient sample, again with a large effect size. In terms of the BE, while patients exhibited a significant decrement, the effect size achieved was only in the medium range (0.67). Correlation analysis suggested that those patients with the weakest BE were those patients also showing the greatest P1-deficit. Coupled with the complete lack of correlation between these metrics in controls, this finding is intriguing. First, the absence of any relationship between P1 amplitude and the BE in controls suggests that separable processes drive them. This dissociability is emphasized by the fact that the P1 and the BE have quite different, albeit overlapping, timecourses. Why then do we find significant correlation in the patients? The implication is that both metrics index a more general set of visual processing deficits in this population. It bears mentioning, however, that data from only 16 patients were entered into this correlation analysis and that replication will be important before strong conclusions can be drawn.

From a diagnostic perspective, it would be highly useful if the P1-deficit and the BE could both be identified as endophenotypic. While evidence is strong that this is so for the P1-deficit, it remains to be seen whether the same is true of the BE. One of our main motivations here stemmed from the notion that by combining these two easily obtained metrics as part of a so-called multivariate endophenotype (15, 41, 42), we might improve classification ability. However, combining P1 and the BE in this relatively small cohort was disappointing, in that the ability of a combined measure to correctly classify patients and controls increased only modestly from 71.9 to 75% when the P1 was used in isolation. Nonetheless, modest classification improvement such as this may yet prove useful when a greater number of endophenotypic measures are combined, especially if measures across sensory modalities are employed (43).

While inter-individual variance of the BE precludes its use as a single clinical marker, it may yet prove useful for future research to parse deficits in basic sensory processing with an intra-individual strategy in mind. For example, since patients with schizophrenia have shown impaired binocular depth perception (25), it could well be the case that a closer examination of the patients in the present study whose modulation index was the lowest would reveal a specific profile of visual sensory impairment that could enable us to begin to tease apart some fundamental trait-level processing deficits. This, in turn, may allow us to classify a sub-group of patients that share specific risk genetics. It may be the case that those patients who failed to show the BE (i.e., had a modulation index of zero or less) are also those individuals who lack stereopsis, although this remains to be explicitly tested. Further, there may be a link between these deficits and deficits of the visual biological motion processing system, which also shows impairments in schizophrenia (44). Linking a cluster of subclinical traits such as these together may prove valuable in allowing us to target the presence of schizophrenia in a prodromal population. Even weakly endophenotypic markers for schizophrenia could still prove useful as elements of a battery of metrics for risk assessment. That is, compilation of a set of measures, some with high sensitivity but low specificity and others with high specificity but low sensitivity, may prove to be an excellent multivariate strategy for assessing risk (41).

There is precedence for manipulating short-term dynamic changes in visual processing as a means to investigate psychiatric illness. For example, Pettigrew and Miller (45) employed binocular rivalry to examine the neuropathology of bipolar disorder [also Ref. (46)]. Binocular rivalry occurs when two different images are presented to each eye simultaneously. Observers perceive either one or the other image alternating between the right and left-eye inputs, and these perceptual switches occur automatically. Pettigrew and Miller found that bipolar patients had slower perceptual switches than controls and they attributed this to what they termed “sticky” inter-hemispheric switches. It is noteworthy that we also found visual P1-deficits in euthymic bipolar patients that mimic those seen in schizophrenia (47), suggesting that the P1-deficit, and binocular interaction anomalies, may result from shared genetic risk for psychotic disorders more generally.

### Potential synaptic mechanisms of binocular interactions and monocular plasticity

In the one existing study to assess short-term visual plasticity in schizophrenia, high-frequency photic inputs were used to enhance/tetanize early visual responsiveness, a paradigm expressly developed to assay putative glutamatergic dysfunction (18). When one considers the potential neurochemical mechanisms of the plasticity revealed by the current paradigm, the bulk of the work on interactions between ocular dominance columns points to primary involvement of the GABAergic system (48, 49) rather than the glutamatergic system, although the picture is surely more complicated than a single transmitter system. The majority of what is known about the effects of MD derives from animal models, almost exclusively involving long-term deprivation preparations (49–53). Early seminal work by Wiesel and Hubel (54) observed that MD in kittens induced a shift in ocular dominance toward the non-deprived eye, an effect also shown subsequently in adult mice (53). By iontophoretically applying the GABA antagonist bicuculline to visual cortical neurons in area V1 of cats monocularly deprived from birth, Burchfiel and Duffy showed that input from the deprived eye could be restored in a large percentage of V1 neurons (48). Their work implied that cells representing the deprived eye were constitutively and strongly inhibited by those representing the non-deprived eye. Work has also suggested that ocular dominance plasticity is dependent on the strength of GABAergic inhibition relative to glutamatergic excitation [reviewed in Ref. (52)]. Mower and Christen (49) showed an “enhanced” role of GABA in animals with abnormal ocular dominance. In cats raised with MD, 50% of visual cortical neurons showed changes in ocular dominance after GABA antagonist administration compared to only 17% of neurons in normal cats.

Here, of course, we used a much shorter-term deprivation paradigm, measured over a matter of minutes rather than hours and days and the effects we observed were fast-acting. One possibility here is that once input is temporarily removed from one eye, that there is a release from inter-ocular inhibition of the other eye, and that the changes we measure in our control populations represent this fast-acting change in GABAergic functioning. By inference then, the lack of a BE would signal GABAergic dysfunction in schizophrenia, and it is of significant interest to note that a relatively recent magnetic resonance spectroscopy (MRS) study reported significantly reduced GABA concentrations in the visual cortex of patients with schizophrenia (55). Yoon and colleagues also showed a significant relationship between MRS measures of GABA and performance levels on an orientation-specific surround suppression judgment task. This task asks participants to determine whether there is a lower contrast target segment in a multi-segment surface and is believed to rely on GABAergic inhibitory surround mechanisms in visual cortex. That patients’ performance on the task was considerably impaired and that performance across patients and control participants was related to GABAergic mechanisms clearly implicates GABA in the visual sensory dysfunctions that are now consistently being observed in schizophrenia. Nonetheless, other possibilities should be considered with regard to the results of the current study, and one of these is that the release from inter-ocular inhibition may also be accompanied by short-term NMDA-based increases in synaptic efficacy within the ocular dominance columns still receiving inputs. Clearly, it will fall to future research efforts to expressly test these possibilities, likely involving direct pharmacological manipulations, and it bears emphasizing that the current study supports only speculations about the underlying synaptic mechanisms since our non-invasive techniques simply cannot directly assess function at this level.

## Study Limitations and Future Considerations

Participants in this study were all receiving anti-psychotic medications at time of testing and one needs to consider the possible effects of such on the recorded VEPs. It would be ideal to replicate these findings in drug-naïve first-episode patients and to assess whether short-term visual plasticity deficits are also present in first-degree biological relatives, which would circumvent this medication issue. However, it is important to point out that the visual sensory processing deficits seen in the VEP of schizophrenia patients (i.e., the P1 amplitude decrement) are also found in both unmedicated patients and their first-degree relatives (8, 9) and the extent of the P1-deficit was not correlated with anti-psychotic dosage levels in a large-cohort patient study (9). As such, we consider it unlikely that the current effects are related to medication status. Another limitation here is the lack of a behavioral correlate with the observed plasticity deficits. As mentioned above, it would be of significant interest to assess whether weakened monocular plasticity relates to the extent of previously observed stereopsis deficits in schizophrenia (25).

Another consideration relates to how potential attentional differences between groups might play a role in the deficits observed here. There is certainly considerable work pointing to attention-related deficits in schizophrenia (56–58) and VEP work has shown that the P1 component is modulated by shifts in spatial attention (59–61). However, differences in attentional deployment seem an unlikely explanation for a number of reasons. First, there was no difference in how patients with schizophrenia performed the target detection task relative to controls, and the checkerboard stimuli themselves were not explicitly attended by either group since the task was to respond to occasionally occurring pictures of animals. Second, while spatial attention can modulate the P1 evoked to more peripherally presented inputs, a number of studies have shown that when inputs are presented to central fixation, modulation of early VEP components as a function of selective attention is not observed (62, 63). The basic notion is that suddenly onsetting visual inputs to the fovea always receive fully elaborated sensory processing, although it bears pointing out that modest modulations of the P1 can be observed when continuous central stimulation routines are employed and covert spatial attention is deployed toward peripheral events (64). Here, however, the fact that patients were successfully completing the central task makes it clear that they were not consistently attending to peripheral space (where nothing was happening), and even if they somehow were, previous VEP work shows that this would not have resulted in a P1 modulation. Finally, while there is evidence for visuospatial attention deficits in patients with schizophrenia, recent work by Hahn and colleagues shows that these deficits only manifest under conditions where relatively complex spatial arrays are used. They used a design where there were four potential peripheral target locations and found that if they spatially cued just one or two locations on a given trial, patients showed the same performance benefits in responding to validly cued targets vs. targets that appeared at uncued locations. However, when the number of cued locations increased to four, the patients now showed deficits in performance relative to controls. In the current study, there was only one location used and as above, there was no requirement to shift spatial attention.

While the P1 is an accepted index of early sensory processing, it could reasonably be asked why an even earlier VEP component, the C1, was not explicitly assayed here. Again, prior work by our group has shown evidence for deficits in this component in patients with schizophrenia. A key aspect of the C1 is that it is known to be generated in early retinotopic cortex (i.e., V1 and V2) since it reverses in polarity dependent on whether visual inputs are presented to the upper or lower visual fields and in its projection to the scalp dependent on whether inputs are presented to the left or right of fixation (65, 66). Here, we used central presentations, which by definition lead to activation of all four quadrant representations of space in area V1. Given the cruciform geometric arrangement of the four V1 quadrants, the general effect is for these four generators to cancel each other out to a large extent as this arrangement leads to a macroscopic “closed field.” As can be seen in the waveforms of Figure 1, there is very little evidence of a C1, as one would expect for our central presentations. Also, the use of the statistical cluster plot method in *post hoc* analysis would have revealed possible effects of the main manipulation of this experiment if they were indeed to be seen during the C1 time-frame, but as can be seen in Figure 5, no effects are evident before 100 ms. A comprehensive investigation of the C1 component in schizophrenia is certainly merited, but such a study would require explicit methods to individually map the retinotopic projections at the individual participant level, a method that has been successfully applied in neurotypical observers (61).

Finally, inspection of the waveforms of Figure 1 reveals a number of interesting between group differences that will merit follow-up in future studies. The reader will note that during the time-frame of the N1 component (circa 120–180 ms), control participants show a biphasic pattern of negative waves, whereas the pattern in patients is monophasic. We have no hypothesis regarding this unanticipated effect, but this difference will clearly merit replication and a fuller exploration in future work. Similarly, the patient waveforms show a distinct negative-going slow potential that appears to be related to some anticipatory mechanisms since it precedes stimulus onset, whereas the controls show little evidence of such anticipatory potentials. The reader will recall that a very wide range of inter-stimulus-intervals was used between instances of the checkerboard stimulus (740–1540 ms). As such, the stimuli were not rhythmic, as is often the case in studies such as this, and it is therefore somewhat surprising to see anticipatory potentials. Given the low probability of a target stimulus and the lack of predictable timing in the sequence, the deployment of anticipatory mechanisms could be considered maladaptive. Nonetheless, patients performed the task at a high degree of accuracy, so their strategy appears to have been effective. Again, it will bear following up in a specifically designed study to see whether patients with schizophrenia deploy anticipatory mechanisms in a sub-optimal fashion. It is key to point out, however, that this negative-going effect does not impact the main comparison of interest in the current study, where our primary hypothesis related to the difference between the monocular and binocular conditions (i.e., the BE). It is clear from Figure 1 that these anticipatory potentials are not driving the differences seen during the P1 time-frame and the subtraction waves of Figure 3 show that the early BE occurs in a much higher frequency band.

## Conclusion

We developed a simple MD paradigm to assay short-term plasticity within early visual cortices during the initial sensory processing time-frame. Our overarching hypothesis was that short-term compensatory mechanisms in the visual system of neurotypical controls would allow for a relatively rapid recovery of VEP amplitude during periods of MD, but that this rapid plasticity would be less effectively engaged in patients with schizophrenia. Controls exhibited a robust BE; that is, when we added the evoked response elicited from viewing with one eye to that of the other eye and compared this sum to the response resulting from stimulation of both eyes together, we observed a non-additive response. Compensatory mechanisms related to ocular dominance plasticity were likely engaged to “boost” the response to inputs from the non-occluded eye. Patients with schizophrenia, on the whole, failed to exhibit this BE, suggesting that the same process of cortical plasticity that allowed controls to generate robust VEPs in the absence of input from both eyes was not appropriately invoked in this population. This simple assay of plasticity in the visual system may serve as a useful endophenotype for schizophrenia in future studies.

## Authors Contribution

John J. Foxe designed the study. Victoria M. Leavitt and Sherlyn Yeap recruited the patients and acquired the data. Victoria M. Leavitt and John J. Foxe analyzed the data. Sherlyn Yeap performed the clinical phenotyping of the patient cohort. John J. Foxe and Victoria M. Leavitt wrote the initial drafts of the manuscript, and all three authors provided editorial input to later drafts. All authors reviewed the final version and have approved it for publication in its present form. All authors had access to the original data and the records of all subsequent analyses.

## Conflict of Interest Statement

The authors declare that the research was conducted in the absence of any commercial or financial relationships that could be construed as a potential conflict of interest.

## Supplementary Material

The Supplementary Material for this article can be found online at http://www.frontiersin.org/Journal/10.3389/fpsyt.2013.00164/abstract

Figure S1 **The centrally presented visual stimuli used in the task**. Event-related potential waveforms were derived for the isolated-check non-target stimulus (A) while target discrimination was performed on the basis of infrequently presented animal line drawings (B) and (C).

Figure S2 **The scatter plot displays P1 amplitude for each participant; averages were taken after collapsing over left and right hemiscalp electrodes**. Controls’ values are shown in blue (*n* = 16), patients in red (*n* = 16). The average of the controls’ P1 amplitude was 6.33 μV; patients’ was 3.34 μV. The effect size of the difference (using Cohen’s *d*) is 1.05.

## Footnotes

^1^In four control participants and four patients, a clear P1 could be identified over only one hemiscalp. The hemiscalp of absent P1 was not consistent across subjects. These subjects were excluded from this P1 2 × 2 ANOVA.
