# Supplementary material for: Brief Monocular Deprivation as an Assay of Short-Term Visual Sensory Plasticity in Schizophrenia – “The Binocular Effect”
Source: Front Psychiatry. 2013 Dec 17;4:164. doi: 10.3389/fpsyt.2013.00164 (PMC3865422; doi:10.3389/fpsyt.2013.00164)
Supplement: Figure S1 — The centrally presented visual stimuli used in the task. Event-related potential waveforms were derived for the isolated-check non-target stimulus (A) while target discrimination was performed on the basis of infrequently presented animal line drawings (B) and (C). [file 62405_Foxe_DataSheet1.DOC]

Brief monocular deprivation as an assay of short-term visual sensory plasticity in schizophrenia – “the binocular effect”.

John J. Foxe, Ph.D. ^1,2,3,4,*^, Sherlyn Yeap, Ph.D., MRCPsych ^1,2^,

Victoria M. Leavitt, Ph.D. ^1,3^

Supplementary Materials

Supplementary Figure 1. The centrally presented visual stimuli used in the task. Event-related potential waveforms were derived for the isolated-check non-target stimulus (A) while target discrimination was performed on the basis of infrequently presented animal line drawings (B and C).


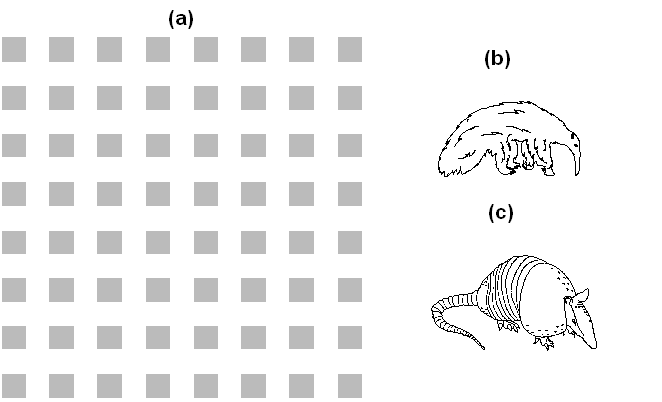


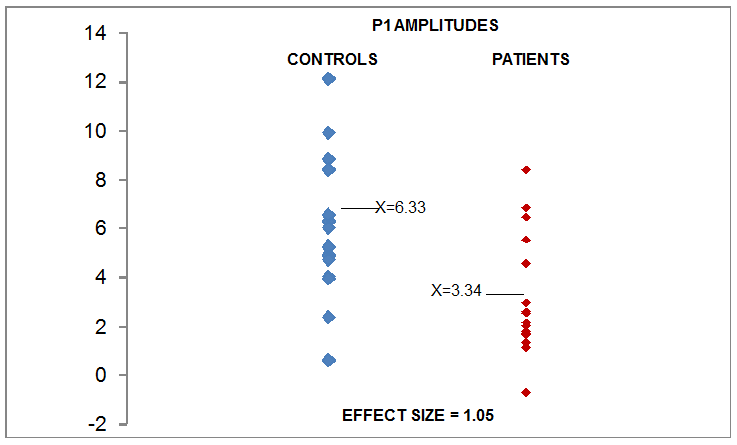
Supplementary Figure 2. The scatter plot displays P1 amplitude for each participant; averages were taken after collapsing over left and right hemiscalp electrodes. Controls’ values are shown in blue (n=16), patients in red (n=16). The average of the controls’ P1 amplitude was 6.33 μV; patients’ was 3.34 μV. The effect size of the difference (using Cohen’s d) is 1.05.
